# Circulating levels of PRO-C3 reflect liver fibrosis and liver function in HIV positive patients receiving modern cART

**DOI:** 10.1371/journal.pone.0219526

**Published:** 2019-07-11

**Authors:** Leona Dold, Mette J. Nielsen, Michael Praktiknjo, Carolynne Schwarze-Zander, Christoph Boesecke, Robert Schierwagen, Raphael Mohr, Jan-Christian Wasmuth, Christian Jansen, Jenny Bischoff, Jürgen Kurt Rockstroh, Morten A. Karsdal, Ulrich Spengler, Jonel Trebicka, Diana J. Leeming

**Affiliations:** 1 Department of Internal Medicine I, University of Bonn, Bonn, Germany; 2 German Centre for Infection Research (DZIF), Partner Site Bonn-Cologne, Bonn, Germany; 3 Nordic Bioscience, Fibrosis Biology and Biomarkers, Herlev, Denmark; 4 Department of Internal Medicine I, University Hospital of Frankfurt, Frankfurt, Germany; 5 European Foundation for the Study of Chronic Liver Failure (EF-CLIF), Barcelona, Spain; 6 Faculty of Health Sciences, University of Southern Denmark, Odense, Denmark; University of Navarra School of Medicine and Center for Applied Medical Research (CIMA), SPAIN

## Abstract

**Background and aims:**

Although combined antiretroviral treatment (cART) has improved overall survival of HIV infected patients, liver fibrosis and liver related-mortality still constitute major challenges in HIV positive patients. Collagen accumulates in the liver during fibrogenesis. Recent studies showed that circulating levels of extracellular matrix (ECM) fragments might reflect degree of portal hypertension and fibrosis stage in liver disease. In this study, we analyzed the correlation between liver fibrosis assessed by Fibroscan and levels of the formation and degradation markers of type III and IV collagen in HIV positive patients receiving cART.

**Methods:**

116 HIV positive patients (82.7% male, median age 47 years) were enrolled into the study. Liver stiffness and liver fat content were determined using a Fibroscan with integrated CAP function. We quantified ECM formation and degradation fragments of collagen III and IV: PRO-C3, PRO-C4, C3M and C4M. These fragments were measured in peripheral serum by using specific ELISAs.

**Results:**

Fifteen (12.9%) out of the 116 HIV positive patients had relevant fibrosis with a liver stiffness ≥ 7.1 kPa, and 79 patients had relevant steatosis with a CAP value > 248 dB/m. Circulating PRO-C3 levels significantly correlated with increasing degree of liver fibrosis assessed by Fibroscan (p = 0.0005), as well as with APRI score (p = 0.015). Interestingly, circulating PRO-C3 levels were significantly correlated with bilirubin (p = 0.022), reduced platelet count (p = 0.0008) and low albumin levels (p = 0.001), suggesting the association of type III collagen deposition with impaired liver function. None of the other measured ECM components significantly correlated with fibrosis or steatosis.

**Conclusion:**

The formation marker of type III collagen, PRO-C3 not only reflects liver fibrosis, but might also mirror liver dysfunction in HIV positive patients receiving cART. Therefore, the circulating levels of PRO-C3 might be suitable to monitor progression of liver fibrosis and deterioration of liver function in HIV positive patients receiving cART.

## Introduction

Combined antiretroviral therapy (cART) has reduced death rates from opportunistic diseases. However, HIV positive patients are at risk to develop non-alcoholic fatty liver disease (NAFLD) and liver fibrosis. Mortality from liver related complications, such as cirrhosis and liver cancer remain a common problem in these patients [1, 2]. Although antiviral treatment usually exerts a positive impact on liver fibrosis [3–5], cART might cause oxidative stress and contribute to liver dysfunction. On the other hand HIV infection itself leads to altered lipid metabolism and bacterial products can accumulate in the portal circulation owing to an impaired gut mucosal barrier [6, 7]. All these factors determine liver injury in HIV positive patients.

During liver fibrosis, extracellular matrix (ECM) proteins are degraded by matrix metalloproteinases (MMPs) into soluble fragments. These fragments can be measured in the patients’ blood and reflect hepatic remodeling processes. Levels of MMP-driven degradation of type III and IV collagen (C3M and C4M) and type III and IV collagen formation markers (PRO-C3 and PRO-C4) have been demonstrated to reflect antifibrotic therapy in bile duct ligated rats [8]. In HIV positive patients, cART attenuates ECM remodeling, so that levels of PRO-C4, C3M, and C4M become significantly reduced in HIV positive patients under cART [9].

Of all these formation markers, PRO-C3 (type III collagen) has been intensively studied in liver diseases. PRO-C3 correlates to fibrosis stage and degree of portal hypertension in HIV/HCV co-infected patients [10]. Another study showed, that PRO-C3 correlates to histologic fibrosis progression in patients with chronic hepatitis C. [11]. So far, the relationship between ECM proteins and hepatic steatosis has not been studied.

For the first time, this study investigated a possible association between markers of extracellular matrix with hepatic fibrosis and steatosis in HIV mono-infected patients on a cART. To this end, we measured PRO-C3, PRO-C4, C3M, and C4M and simultaneously assessed fibrosis and steatosisby transient elastography, on a Fibroscan with integrated controlled attenuation parameter (CAP).

## Patients and methods

### Patients

In 2013 we initiated a pilot study at the outpatient department at the Bonn University clinic. 142 patients of the Bonn HIV cohort were recruited. 18 and 7 patients had chronic HCV or HBV infection, respectively and were therefor excluded from the analysis. In one patient, ELISA for determination of ECMs was not successful, leaving 116 patients for the final analysis. All enrolled patients received cART for at least one year before study entry.

Alcohol consumption was evaluated by questionnaire. Men with an alcohol consumption above 30 g/week and women with a consumption above 20g/week were excluded from the study.

Antiretroviral therapy had been started according to the guidelines recommended by the European AIDS clinical society (EACS).

Blood counts and liver-function tests (alanine and aspartate aminotransferases, alkaline phosphatase, bilirubin, and y-glutamyltranspeptidase) were determined by routine biochemical procedures. All patients underwent clinical examination, Fibroscan and CAP measurement. Fibroscan with CAP was performed at the same day as blood samples were taken for all biochemical analyses.

Before study inclusion, written consent for all study procedures was obtained from all participants. The local ethics committee of the University of Bonn (decision number 069/10 and 197/11) approved the study. All study procedures were in agreement with the 1975 Declaration of Helsinki. The study was supported by a grant from the German center for infection research (DZIF).

### Enzyme-linked immunoabsorbent assay

Markers of type III and IV collagen formation (PRO-C3 and PRO-C4) and degradation (C3M and C4M) were assessed by specific enzyme-linked immunosorbent assays (ELISAs) in patient serum samples as described elsewhere [12–15].

### Diagnosis of HIV and viral hepatitis

HIV viral load was determined quantitatively with the Abbott RealTime m2000rt (Abbott Laboratories, Illinois, USA). The assay had a detection limit of 40 copies/mL. Chronic viral hepatitis was ruled out by routine assays for hepatitis B surface antigen, HBV-DNA, HCV antibodies and HCV-RNA.

### Transient elastography and CAP measurement

Liver stiffness was measured with a “Fibroscan 502 Touch” device equipped with an M and XL probe (Echosens, Paris, France). In patients with obesity (BMI >30 kg/m^2^) the XL probe was used. We assessed hepatic steatosis via the controlled attenuation parameter (CAP) technology [16, 17]. CAP measures the attenuation of elastic waves in liver tissue at the center frequency of the Fibroscan probe and yields results in dB/m, which correspond to intrahepatic fat content. Liver stiffness results are expressed in kPa. Liver stiffness and CAP were obtained simultaneously in the same volume of liver parenchyma and constitute the median of 10 measurements. Patients had to be in fasting condition at least 3 hours from the last meal before measurement.

In order to identify patients with fatty liver disease by CAP we used the criteria published by Karlas [18, 19]. Thus, patients were classified as having no steatosis when CAP values were ≤248 dB/m and as having steatosis when CAP values were higher than 248 dB/m.

Liver stiffness was allocated to histological Metavir fibrosis scores with the following thresholds as defined by Castera et al. [20, 21]. A Stiffness ≥7.1 kPa was classified as relevant fibrosis (Metavir F≥ 2) and a stiffness of ≥12.5 kPa (Metavir F≥3–4) was considered as severe fibrosis.

### Statistical methods

ECM levels were compared between patient groups and controls by Pearson’s goodness-of-fit chi^2^ test. Differences between groups were analyzed by t-test and ANOVA.

Receiver-operating characteristics curves were constructed to assess the accuracy of PRO-C3 measurement and to determine the optimal cutoffs to differentiate between relevant or non-relevant fibrosis (cutoff 7.1 kPa). The optimal cutoff value of PRO-C3 was chosen at the point with the highest Youden’s index. Correlations between ECM levels, liver stiffness determined by Fibroscan and biochemical data were calculated by using Spearman correlation coefficient. A Logistic regression analysis was calculated in order to identify factors associated with relevant fibrosis (liver stiffness ≥7.1 kPa) or steatosis (CAP >248 dB/m).

APRI score was calculated in all patients as previously described [22].

All statistical tests were performed and figures created by using GraphPad PRISM version 7 (GraphPad Software, La Jolla California USA) and SPSS 22.0 software (SPSS, Munich, Germany).

## Results

### Patient characteristics

116 HIV-infected patients were enrolled into this study. All patients received cART for a median of 72 months at date of enrollment (range 12–216 months). The median age of all participants was 47.3 years (range 23.9–71.9).

**Fig 1:**
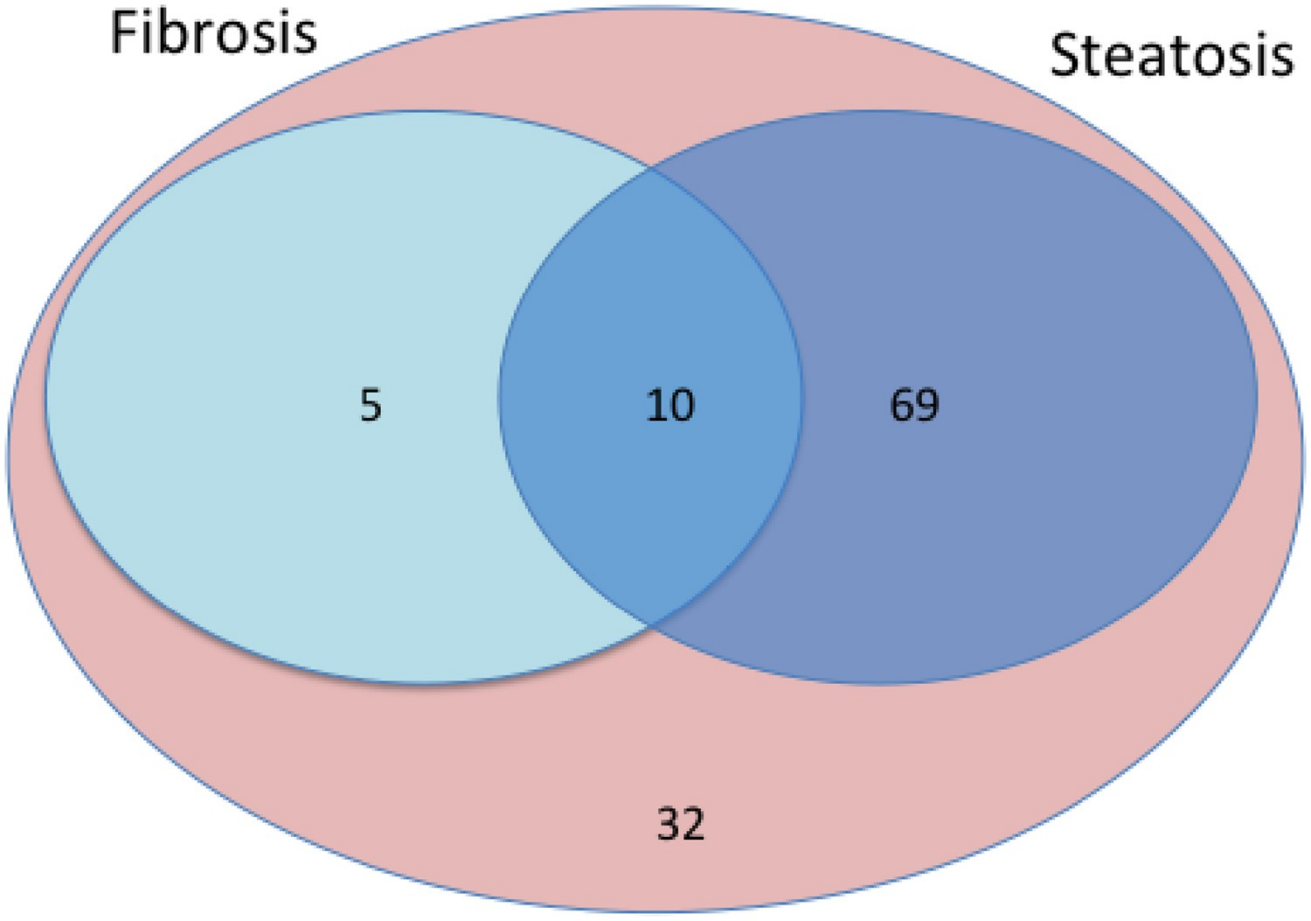
Study cohort. The Venn-diagram depicts the number of patients with relevant fibrosis (n = 5) or relevant steatosis (n = 69 patients) or both (n = 10), measured by Fibroscan. Thirty-two patients had neither relevant fibrosis, nor steatosis. The total of 116 patients was included into the study.

Patients were divided into two groups according to their Fibroscan stiffness (Table 1). Fifteen (13%) patients had relevant hepatic fibrosis F2 (liver stiffness ≥7.1 kPa) and 101 patients had no relevant fibrosis (liver stiffness <7.1 kPa). Based on their CAP results, 79 HIV-positive patients (68%) were allocated to have fatty liver disease (CAP >248 dB/m) (Table 2). In 37 patients (32%) no hepatic steatosis was measured (CAP ≤248 dB/m). Patient characteristics are summarized in Tables 1 and 2. Distribution of patients is demonstrated in the Venn diagram (Fig 1), showing that 32 patients in the cohort had no relevant fibrosis or steatosis.

**Table 1:** Clinical parameters of patients stratified by stiffness. All values are given as median and range. BMI: Body-mass index, AST: aspartate aminotransferase, ALT: alanine aminotransferase; GGT: γ–glutamyl transferase, CAP: Controlled attenuation parameter.

	HIV (all)	HIV patients without significant fibrosis (Stiffness <7.1 kPa)	HIV patients with significant fibrosis (Stiffness ≥7.1)	P-value
**Total number of patients n (%)**	116 (100)	101 (87.1)	15 (12.9)	n.s
**Age (years)**	47.3 (24–72)	46.5 (24–72)	53.9 (45–67)	**0.001**
**Gender (male/female)**	96/20	85/16	11/4	n.s
**BMI (kg/cm**^**2**^**)**	23 (17–61)	23 (17–61)	25 (19–30)	n.s
**AST (U/L)**	23 (9–93)	23 (9–65)	26 (16–93)	**0.002**
**ALT (U/L)**	29 (10–99)	29 (10–84)	31 (13–99)	n.s
**GGT (U/L)**	45 (17–302)	45 (17–302)	55.0 (26–228)	n.s
**Bilirubin (mg/dl)**	0.45 (0.15–4.5)	0.45 (0.15–4.6)	0.39 (0.27–2.3)	n.s
**Stiffness Fibroscan (kPa)**	4.8 (2–14.8)	4.6 (2.0–6.8)	8.2 (7.1–14.8)	**<0.001**
**Steatosis CAP (dB/m)**	233.5 (100–378)	231.0 (100–378)	243.0 (101–351)	n.s
**APRI Score**	0.2 (0.1–1.4)	0.2 (0.1–0.9)	0.3 (0.2–1.4)	**<0.001**
**PRO-C3 (ng/ml)**	9.8 (5–40)	9.4 (5–24)	13.8 (8.-40)	**<0.001**
**PRO-C4 (ng/ml)**	274 (126–1023)	262 (126–1023)	309.9 (128–510)	n.s
**C3M (ng/ml)**	8.6 (5–20)	8.6 (5–20)	9.1 (7–17)	n.s
**C4M (ng/ml)**	57.5 (30–130)	56.9 (30–123)	63.5 (39–130)	n.s

**Table 2:** Clinical parameters stratified by CAP values. All values are given as median and range. BMI: Body-mass index, AST: aspartate aminotransferase, ALT: alanine aminotransferase;; GGT: γ–glutamyl-transferase, CAP: Controlled attenuation parameter.

	HIV (all)	HIV patients without fatty liver (CAP ≤ 248 dB/m)	HIV patients with fatty liver (CAP > 248 dB/m)	p-value
**Total number of patients n (%)**	116 (100)	79 (68)	37 (32)	n.s
**Age (years)**	47.3 (23.9–71.9)	46.5 (23.9–71.9)	49.3 (30.4–67.0)	n.s
**Gender (male/female)**	96/20	63/16	33/4	n.s
**BMI (kg/cm**^**2**^**)**	23.3 (17.3–60.6)	23.0 (18.5–60.6)	24.3 (17.3–35.5)	n.s
**AST (U/L)**	23 (9–93)	23 (9–93)	24 (13–65)	n.s
**ALT (U/L)**	29 (10–99)	29 (10–99)	31 (16–84)	n.s
**GGT (U/L)**	45 (17–302)	44 (17–301)	53 (22–302)	n.s
**Bilirubin (mg/dl)**	0.45 (0.15–4.5)	0.42 (0.15–3.05)	0.48 (0.21–4.6)	n.s
**Stiffness Fibroscan** **(kPa)**	4.8 (2–14.8)	4.6 (2.0–14.8)	5.3 (3.2–9.3)	n.s
**Steatosis CAP (dB/m)**	233.5 (100–378)	218 (100–248)	294.0 (252–378)	**<0.001**
**APRI Score**	0.2 (0.1–1.4)	0.2 (0.1–1.4)	0.3 (0.1–0.9)	n.s
**PRO-C3 (ng/ml)**	9.8 (5.46–39.5)	9.4 (5.5–39.5)	10.4 (5.5–23.6)	n.s
**PRO-C4 (ng/ml)**	274 (126.4–1023.4)	263.0 (126.4–573.5)	297.8 (147.9–1023.4)	n.s
**C3M (ng/ml)**	8.6 (4.8–19.5)	8.5 (4.8–14.2)	9.6 (5.6–19.5)	**0.046**
**C4M (ng/ml)**	57.5 (29.8–130.1)	55.3 (29.8–88.9)	60.7 (37.7–130.1)	**0.039**

Patients with relevant fibrosis were significantly older (p = 0.001) and had higher AST levels (p = 0.002), higher APRI scores (p< 0.001) and, higher PRO-C3 levels (p<0.001).

HIV positive patients with hepatic steatosis (CAP >248 dB/m) had no significant differences in clinical parameters as compared to patients without steatosis. However, levels of C3M and C4M were significantly higher in patients with steatosis (p = 0.046 and p = 0.039, Table 2).

The median cART duration at study entry was 92.9 months and patients received cART at least since one year before study entry. Seventy-two (36.2%) patients were previous treated with non-nucleoside reverse transcriptase inhibitors (NNRTI).

74 (37.2%) with protease inhibitor- based antiretroviral regimes and twenty-two (11.1%) patients had a history of D-drugs (e.g. didanosine and stavudine).

Patients with prior or current use of protease inhibitors showed higher serum bilirubin levels (0.786 versus 0.355; p<0.001) than patients without PI exposure. An increased bilirubin, however, was not statistically associated to the use of Atazanavir. The distribution of antiretroviral regimens did not differ significantly between HIV positive patient groups with relevant hepatic steatosis and/or fibrosis and the levels of ECMs were not attributed to a specific substance class.

### Levels of PRO-C3 reflect liver function and liver fibrosis measured by Fibroscan

**Fig 2:**
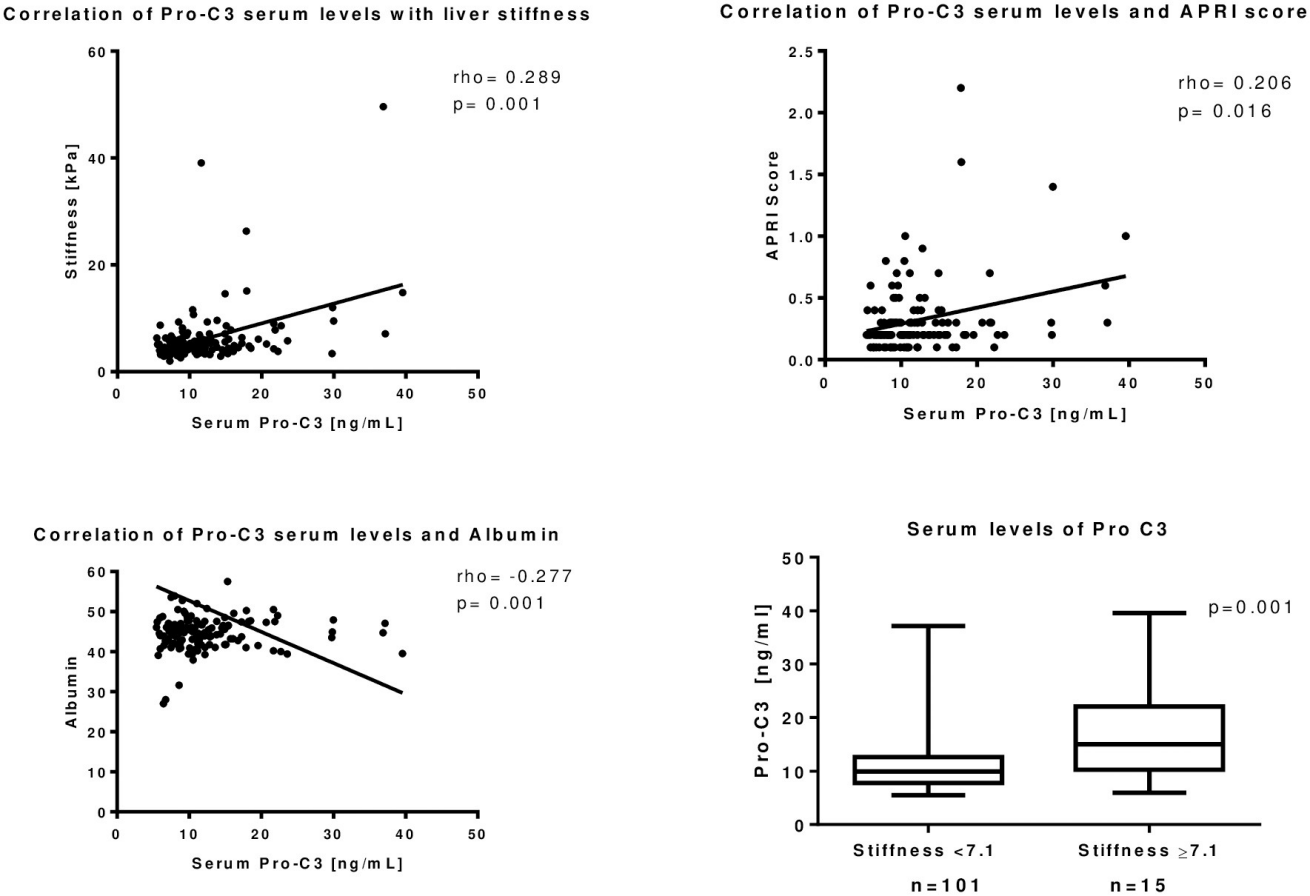
Levels of PRO-C3 measured in blood of patients with HIV infection correlated with liver stiffness and liver function parameters. Levels of PRO-C3 correlated with liver stiffness measured by A) Fibroscan (rho = 0.289; p = 0.001) and B) APRI score (rho = 0.206; p = 0.016) and inversely with C) albumin (rho = -0.277; p = 0.001). Furthermore, D) significant higher levels of PRO-C3 could be detected in patients with relevant fibrosis versus patients without fibrosis (p = 0.001).

Levels of serum PRO-C3 significantly associated with liver fibrosis measured by Fibroscan (rho = 0.289; p = 0.0005) (Fig 2A), bilirubin (rho = 0.196; p = 0.022) and APRI score (rho = 0.206; p = 0.0156) (Fig 2B). PRO-C3 inversely correlated with levels of cholinesterase (rho = -0.2401; p = 0.0052), platelet counts (rho = -0.279; p = 0.0008) (S1 Table) and albumin (rho = -0.277; p = 0.001) (Fig 2C). and parameters of liver function. Levels of serum PRO-C3 were significantly higher in patients with a Fibroscan stiffness of ≥7.1 kPa (Fig 2D).

In order to further determine the significance of PRO-C3 for the detection of a relevant liver fibrosis ≥7.1 kPa a Logistic regression was calculated. Regression analysis confirmed PRO-C3 levels as independent risk factor for relevant fibrosis (p = 0.003) together with AST levels (p = 0.038) (Table 3). All other factors (Age, BMI, APRI score, ALT, yGT, Bilirubin, PRO-C4, C3M and C4M) did not remain statistically significant.

**Table 3:** Regression analysis for independent risk factors. AST: aspartate aminotransferase.

Regression analysis Risk factors for fibrosis (≥7.1 kPa)			
**Parameter**	**OR**	**95%-CI**	**p-value**
AST/GOT	1.049	1.003–1.097	0.038
PRO-C3	1.207	1.065–1.367	0.003
Regression analysis Risk factors for steatosis (>248 dB/m)			
**Parameter**	**OR**	**95%-CI**	**p-value**
Triglycerides	1.005	1.002–1.009	0.004
C3M	1.229	1.013–1.491	0.037

Using Receiver-operating characteristics curves we identified a PRO-C3 value of 15 ng/ml as cutt-off, to stratify patients with a liver stiffness of ≥7.1 kPa in transient elastography (AUC 0.729 p = 0.010) and calculated a Sensitivity of 36.4% and a Specificity of 88,5% (positive predictive value 25%, negative predictive value 93%) for this cut-off.

In order to rule out bias from false-positive results caused by obesity, we re-calculated our dataset, and excluded patients with a BMI >30 kg/m^2^ (n = 14). Than, Pro C3 at 15 ng/ml had only a slightly superior performance (AUC 0.746 p = 0.006) with a Sensitivity of 40.0% and a Specificity of 90.0% (positive predictive value 33%, negative predictive value 92%).

### Relationship between C3M, C4M and PRO-C4 levels and results of stiffness and CAP measurements

**Fig 3:**
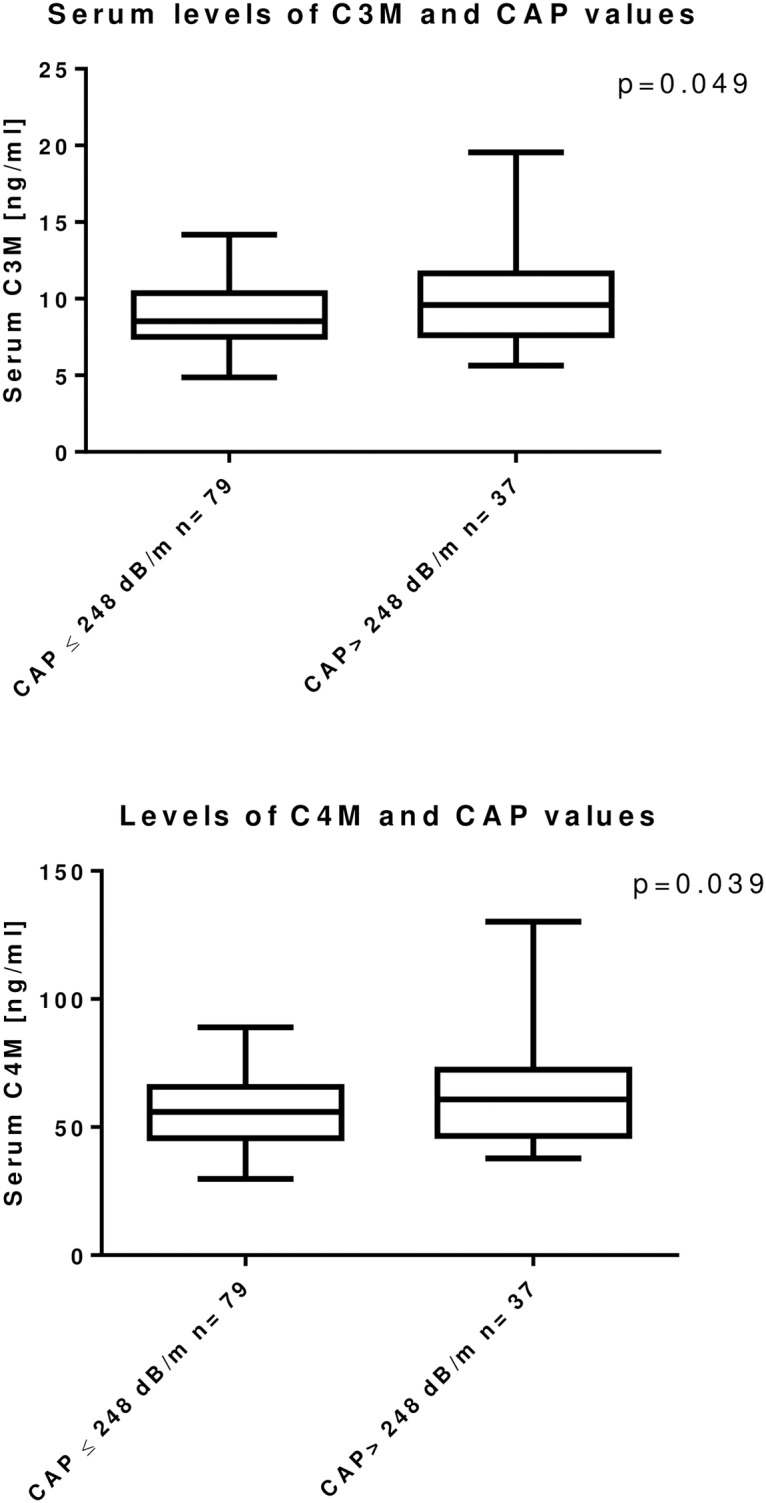
Levels of C3M and C4M in the blood of HIV positive patients correlates with steatosis measured by CAP. In patients with an increased liver fat content, higher C3M levels (p = 0.046) and higher C4M levels (p = 0.039) were detected in blood samples.

Serum C3M levels were correlated to steatosis as determined by CAP measurement (rho = 0.192; p = 0.022) (Fig 3 and S1 Table). We further calculated a Logistic regression analysis in order to identify risk factors for relevant steatosis (CAP >248 dB/m) and C3M was confirmed as an independent risk factor (p = 0.037) together with triglycerides (Table 3).

When we stratified the patient cohort by their CAP values (Table 2), the difference in C3M and C4M levels was significant between patients with CAP values below or above 248 dB/m (Fig 3), but a significant impact of C4M was neither confirmed by Spearman correlation, nor by Logistic regression. For PRO-C4 no correlation to fibrosis or steatosis in Fibroscan measurement was observed.

## Discussion

This study was designed to examine the association between the ECM remodeling markers PRO-C3, PRO-C4, C3M, C4M and hepatic fibrosis and steatosis in HIV positive patients. For the first time, serum levels of ECMs were compared to fibrosis and steatosis determined by Fibroscan in an HIV mono-infected cohort receiving modern cART.

HIV positive patients are at a high risk for liver related complications and elevated liver enzymes [1, 23, 24]. Liver disease has emerged as a severe challenge and contributes to 7%–14% of acquired immune deficiency syndrome (AIDS)- related deaths [2]. Therefore, HIV positive patients benefit from noninvasive screening methods that help to easily identify changes in liver stiffness or liver fat content, as liver biopsy is hampered by sampling error and complications for the patient such as bleedings, pain and hospitalization [25, 26].

Our findings show that PRO-C3 levels are associated with grade of fibrosis in Fibroscan in HIV mono-infected individuals. Furthermore, PRO-C3 correlates to APRI score and parameters of liver function such as bilirubin, cholinesterase, albumin and platelet count.

The fact that Fibroscan is a well-established tool for the non-invasive detection of fibrosis [21, 27], the correlation to PRO-C3 levels may allow to draw a conclusion to Fibroscan values when only PRO-C3 measurements are available.

We calculated that PRO-C3 levels of above 15 ng/ml allows to rule out relevant liver fibrosis, PRO-C3 had a very high negative predictive value and is easy to use and therefore might be of interest for clinical use; especially in primary care centers, where Fibroscan is not available. PRO-C3 might allow to screen and identify patients with a significant liver fibrosis, so that these patients can be referred to a specialized center for further diagnosis.

Our study confirms results from a previous study in a cohort of HCV mono-infected patients, that PRO-C3 is a useful tool to predict levels of liver fibrosis (11). Furthermore, elevated PRO-C3 might serve as a predictor, to identify patients with a beginning acute decompensation of chronic liver injury as described for other liver-diseased patient cohorts [28]. This is an important point in HIV positive patients, who are constantly exposed to potentially hepatotoxic drugs [29] especially in the HAART era as compared to modern cART [30].

Interestingly, our HIV mono-infected patients showed lower PRO-C3 levels as compared to previously reported patient cohorts [10, 11]. However, this is not surprising considering only 15 of 116 patients showed significant hepatic fibrosis. Moreover, in our cohort, HIV viral load was below the detection limit, which represents the nature of most real life cohorts in todays era of modern cART [31]. These facts are suggesting relatively good liver-related condition with little fibrogenesis and hepatic inflammation. This is also reflected by the fact that median ALT and AST levels of our patients are within the normal range, while previously reported HIV/HCV co-infected patients and viremic HCV mono-infected patients had significantly higher levels [10]. This might explain the lower PRO-C3 levels, which suggest less fibrogenesis compared to the co-infected patients with replicative HCV infection. This accelerated inflammation, caused by HCV, is also reflected by the elevated aminotransferases levels, which were also detected in a HCV cohort (11). Our data suggest, that patients with highly controlled HIV infection have PRO-C3 levels that are comparable to a normal healthy population and therefore lower rates of ECM remodeling and subsequent lower risk of fibrosis progression.

Cut-offs for the detection of relevant fibrosis should be adapted depending on the patients condition (HCV mono-, HIV mono- or HIV/HCV co-infection).

We would suggest a PRO-C3 value of 15 ng/ml to rule out significant fibrosis in HIV-monoinfected patients. However, our data are based on Fibroscan as reference standard and not on liver biopsy. Fibroscan cut-offs were mainly derived from non-HIV cohorts and probably from patients with higher fibrosis/steatosis prevalence and therefore the diagnostic performance in HIV cohorts is still not entirely clear and must be further evaluated in larger HIV cohorts.

Our study could confirm previous studies, which could not find any correlation between C3M and fibrosis [11]. However, we detected a trend of C3M elevation in patients with steatosis. Praktiknjo et al. detected a decrease in C3M in acute decompensation of liver cirrhosis [28]. Therefore, C3M might reflect beginning liver dysfunction in patients with progressing steatosis. The elevation of C4M in HIV patients with steatosis remains only weak, as it could not be confirmed by Logistic regression analysis. C4M was identified as an independent predictor of survival in female patients with liver cirrhosis and was positively correlated with immune activation [32]. A role for C4M in hepatic steatosis was not described jet and must be further evaluated in a larger cohort of patients with hepatic steatosis.

This study has several limitations. Its cross-sectional design does not allow to evaluate the dynamics of ECM remodeling and additional risk factors of severe hepatic fibrosis and steatosis over a period of time. The study was not randomized and represents a real-world patient cohort. Additional important analysis, such as Homeostasis model assessment index for insulin resistance or magnetic resonance tomography imaging to quantify body composition, were not available. A Liver biopsy, which still represents the gold standard for the assessment of hepatic steatosis, was not performed. However, the acceptability and potential risks of biopsy make its application difficult for a clinical study.

In conclusion, this study demonstrates that liver fibrosis and liver injury are reflected by PRO-C3 levels in HIV infected patients and is able to rule-out hepatic fibrosis in these patients. Moreover, PRO-C3 may be used to monitor patients at risk of fibrosis in centers where Fibroscan is not available. A combination of both transient elastography and PRO-C3 measurement might lower sample error and can strengthen Fibroscan results.

## Supporting information

S1 Table Correlation table. Data are presented as Spearman Rho and p-value. AST: aspartate aminotransferase, ALT: alanine aminotransferase; CHE: cholinesterase.
(PDF)
