# Supplementary material for: Circulating levels of PRO-C3 reflect liver fibrosis and liver function in HIV positive patients receiving modern cART
Source: PLoS One. 2019 Jul 11;14(7):e0219526. doi: 10.1371/journal.pone.0219526 (PMC6622522; doi:10.1371/journal.pone.0219526)
Supplement: S1 Table — Data are presented as Spearman Rho and p-value. AST: aspartate aminotransferase, ALT: alanine aminotransferase; CHE: cholinesterase. (PDF) [file pone.0219526.s001.pdf]

	Albumin	AST	ALT	Billirubin	CHE	Platelets	APRI	Steatosis assessed by CAP	Fibrosis assessed by TE
PRO-C3	-0.277<br>***0.0010***	0.120<br>0.1570	0.053<br>0.5297	0.196<br>***0.0222***	-0.2401<br>***0.0052***	-0.279<br>***0.0008***	0.206<br>***0.0156***	0.091<br>0.285	0.289<br>***0.0005***
PRO-C4	-0.036<br>0.6711	-0.021<br>0.8054	-0.083<br>0.3286	-0.024<br>0.7845	0.174<br>0.0448	0.013<br>0.8821	-0.067<br>0.4384	0.091<br>0.281	0.084<br>0.3230
C3M	-0.063<br>0.4580	0.020<br>0.8137	-0.038<br>0.6537	0.048<br>0.5826	0.141<br>0.1034	-0.032<br>0.7093	0.004<br>0.9674	0.192<br>***0.022***	0.133<br>0.1153
C4M	-0.167<br>0.0501	0.108<br>0.2005	0.010<br>0.9035	-0.063<br>0.4666	0.132<br>0.1298	0.011<br>0.9002	0.080<br>0.3504	0.130<br>0.125	0.149<br>0.0770
